# Delayed Medical Care Following an Alligator Bite: A Case Report and Literature Review

**DOI:** 10.7759/cureus.53005

**Published:** 2024-01-26

**Authors:** Roksana Hesari, Thaddeus Chuchla, Angelica R Carnemolla, Nicholas Tyndall, Randy Scott

**Affiliations:** 1 Osteopathic Medicine, Lake Erie College of Osteopathic Medicine, Bradenton, USA; 2 Family Medicine, Lake Erie College of Osteopathic Medicine, Bradenton, USA

**Keywords:** literature review, case report, animal-inflicted injuries, reptile bites, wildlife-related injuries, alligator bite

## Abstract

Alligator bites in humans present a significant concern for public safety in the southern United States, especially in states like Florida with substantial alligator populations. Although these reptiles play a vital role in the local ecology, encounters with humans can lead to severe injuries and even fatalities. A case report is presented of a 58-year-old male who suffered an alligator bite while attempting to take a selfie with the reptile during a hunting trip in rural Florida. The patient's injuries included multiple lacerations on the dorsum of his right hand. Despite the incident, the patient hesitated in seeking medical attention due to a lack of insurance, emphasizing the need for public awareness of alligator bite management. The discussion highlights the potential complications of alligator bites, including hemorrhage and infection, as well as the importance of appropriate medical treatment, including wound irrigation, debridement, and antibiotic therapy. Moreover, preventive strategies are discussed, such as maintaining a safe distance from alligators and refraining from feeding them, to ensure coexistence between humans and these reptiles in their natural habitats. As knowledge of alligator bites remains limited, this case report contributes valuable information to promote public safety and guide future research in this area.

## Introduction

In the United States, encounters with crocodilian species, including the American alligator and the American crocodile, have become a subject of concern due to the potential for serious or fatal injuries in humans. These reptiles are capable predators, and their presence is particularly notable in the southern states, including Florida, Georgia, and Louisiana [1,2]. Despite being formidable carnivores with an impressive territorial range, these reptiles hold significant ecological importance, contributing to the balance of the Everglades ecosystem. In fact, strict protection measures are in place to conserve these reptiles, such as the Florida Fish and Wildlife Conservation Commission which regulates the harvesting of wild alligators [3]. Due to these measures, the population of alligators in the southern states has been thriving since 1987, resulting in their removal from the endangered species list. However, the resurgence of the species has raised concerns regarding human safety [4]. Moreover, due to a recent surge in human population growth along the coastal regions of the southern United States, the likelihood of interactions between humans and alligators has emerged as a tangible concern [5]. Notably, the literature has documented a significant increase in unfavorable encounters and the reporting of nuisance complaints involving these reptiles [2].

According to a study spanning the years 1928 to 2009, there were 567 cases of adverse encounters with alligators in the United States [6]. Although these numbers indicate that alligator attacks on humans are infrequent, the consequences can be severe, ranging from minor injuries to amputations and fatalities, particularly when the alligators that are encountered are larger in size. Alligator attacks often occur when humans unintentionally encroach upon alligator habitats, provoking heightened territorial behaviors. Additionally, some of these interactions have been attributed to actions such as illegal feeding or discarding of food near water bodies, leading to a desensitization of alligators to the presence of humans [7].

To address the need for a comprehensive understanding of adverse interactions with crocodilian species, this case report aims to present a unique incident involving a patient who sustained an alligator bite. While the literature on alligator attacks in the United States remains limited, this report draws upon available resources and guidelines from wildlife officials to shed light on the epidemiology, pathophysiology, and management of such encounters. By exploring this specific case, our objective is to contribute to the knowledge base surrounding alligator bites, promoting public safety, and guiding future preventive strategies.

## Case presentation

A 58-year-old right-hand dominant Caucasian male presented to an outpatient family medicine office for evaluation of several lacerations on his right hand. The patient reported that he was bitten by an alligator while attempting to take a photograph with the reptile during a hunting trip in rural Florida seven days ago. The incident occurred while the patient was intoxicated, having consumed a six-pack of beer that day. As a result of the encounter, the dorsum of his right hand sustained multiple lacerations. Due to a lack of medical insurance, the patient hesitated for seven days before seeking medical evaluation at the clinic. Upon scheduling an appointment, his sole complaint was the need for stitches. The patient denied any fevers, chills, numbness, tingling, or any additional complaints.

**Figure 1:**
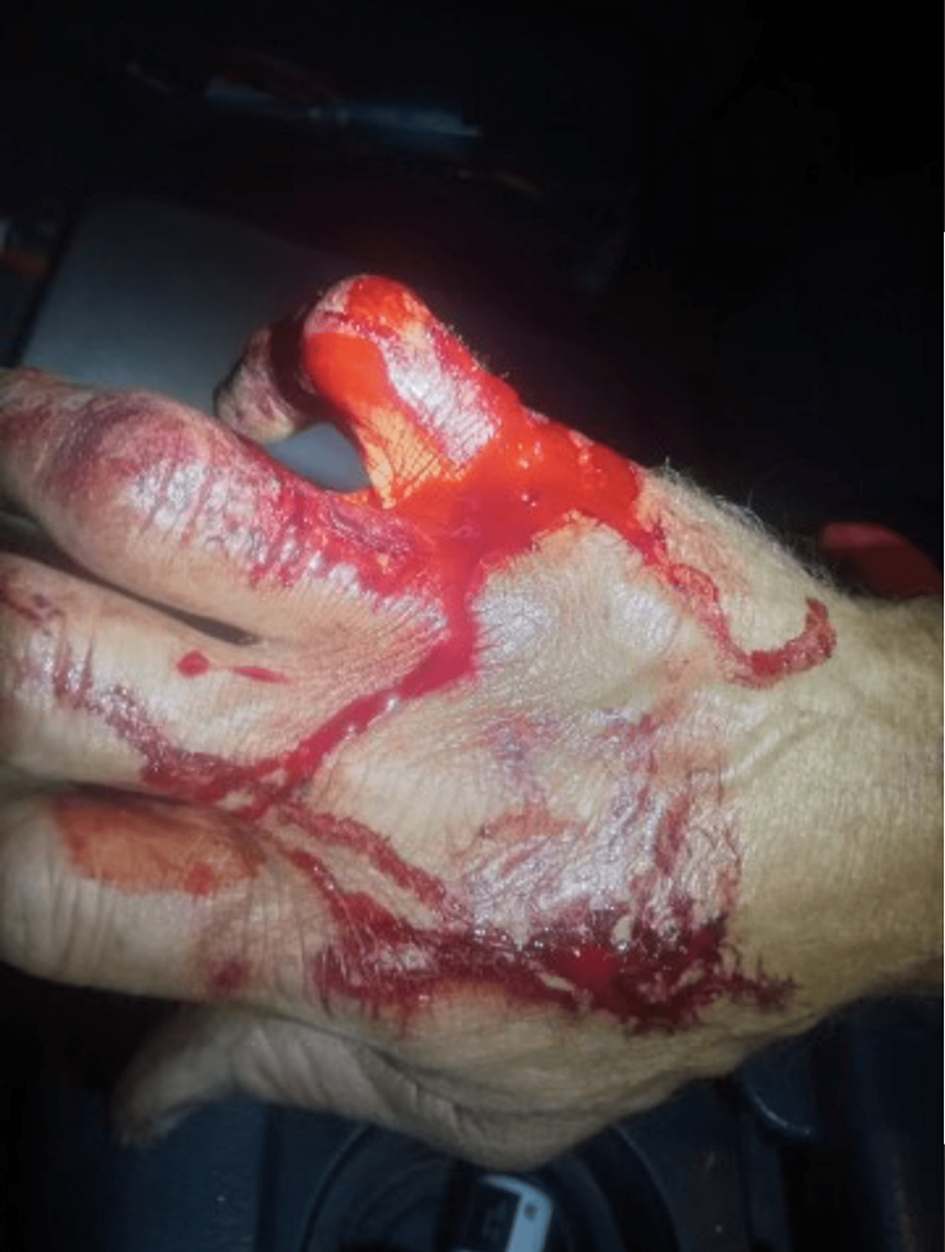
Hand trauma immediately after alligator bite

A laceration measuring 1.8 cm was observed on the dorsum of the right hand, positioned between the fourth and fifth metacarpals. Additionally, situated just above the aforementioned laceration were two puncture marks, each around 0.4 cm in diameter. Moreover, on the lateral dorsum of the right hand, a separate laceration measuring 1.0 cm in length, was discernible. The wounds appeared to be distinct, manifesting as a pattern of superficial and finely incised marks upon the skin surface. The presence of blood surrounding the impacted area pointed towards the recent trauma inflicted by the alligator bite. Figure 1 displays an image showcasing the patient's initial injuries for reference.

Although antibiotics were extended as an option for infection prevention and an X-ray was strongly advised to unveil any potential underlying damage, the patient opted to forego both courses of treatment. Subsequent to the evaluation, the patient was discharged with instructions to follow up in a week and to come sooner if he experienced any alterations in the wound's condition or the onset of any additional symptoms.

**Figure 2:**
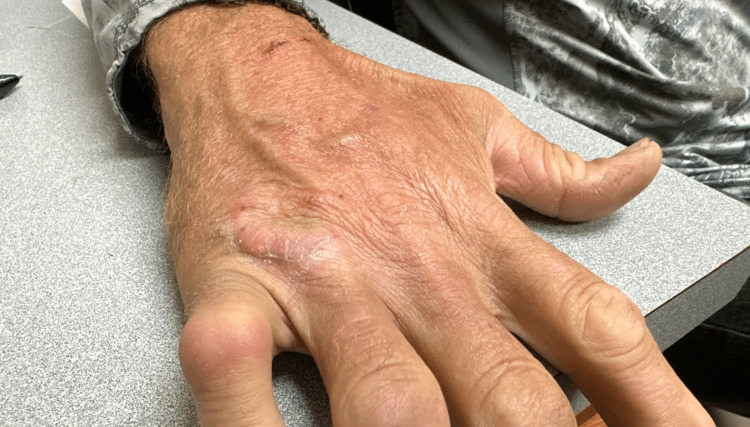
Hypertrophic scar on the dorsum of the right hand, overlying the fourth metacarpophalangeal joint, eight days following the presentation to the clinic.

Eight days later, the patient revisited the clinic for a follow-up appointment. His hand wounds exhibited satisfactory healing, showing no indications of infection or any compromise in hand functionality, as shown in Figure 2.

## Discussion

Alligator bites can result in a range of injuries, including lacerations, puncture wounds, and abrasions. The severity of these injuries, stemming from the significant bite force and subsequent risk of infection, could potentially lead to amputations or the loss of life [8]. The immediate complication most frequently encountered after an alligator bite is hemorrhage, demanding swift control through direct pressure on the wound, followed by wound packing [8]. If left untreated, a cascade of severe complications may ensue, including bacterial infections, tissue necrosis, compartment syndrome, and systemic reactions.

Initially, the treatment of alligator bites often involves wound irrigation, followed by extensive debridement and subsequent wound cultures [9]. Open wounds may be treated with delayed primary closure or may be allowed to heal by secondary intention [8,9]. Standard anti-tetanus measures, involving either a Tdap or Td vaccine, are routinely administered. In some cases, immunoglobulin may be administered along with prophylactic broad-spectrum antibiotics, dependent upon the patient’s immunization history and wound severity [9,10].

The oral microbiome of alligators harbors an array of pathogens that can precipitate infections in bite victims. Specifically, Aeromonas hydrophila, an aerobic gram-negative rod, stands out as one of the most prevalent microbes cultured in such scenarios [8,9,11]. A detailed understanding of the pathogen spectrum is crucial for effective pharmacologic treatment and curbing antibiotic resistance; thus, Table 1 lists the pathogens encountered in the oral microbiota of alligators indigenous to the southeastern region of the United States. Empiric antibiotic therapy typically targets gram-negative microbial species, given their prevalence, although consideration of the less common pathogens is also imperative [11,12]. To ensure comprehensive coverage, recommended antibiotics encompass third-generation cephalosporins and aminoglycosides, administered individually or in combination [9,11]. Table 2 delineates the suggested treatments for the most frequently encountered pathogens [11,13,14]. While empiric treatment holds significance, wound cultures are essential to ascertain the most effective pharmacologic interventions and mitigate the risk of antibiotic resistance.

**Table 1:** Common pathogens found in the oral flora of alligators found in southeastern US [11,12]

Pathogens in Order of Frequency	Class
Aeromonas hydrophila	Aerobic Gram-negative rod
Proteus vulgaris	Aerobic Gram-negative rod
Clostridium bifermentans	Anaerobic Gram-positive rod
Bacteroides bivius	Anaerobic Gram-positive rod
Citrobacter freundii	Aerobic Gram-negative rod
Rhodotorula rubra	Fungi - Microbotryomycetes

**Table 2:** Recommended antimicrobials for treatment of pathogens associated with alligator bites

Pathogen	Recommended Antimicrobial
Aeromonas hydrophila	Third-generation Cephalosporin, Aminoglycoside, Ticarcillin/Clavulanate, Trimethoprim-Sulfamethoxazole
Proteus vulgaris	Third-generation Cephalosporin, Trimethoprim-Sulfamethoxazole
Clostridium bifermentans	Clindamycin, Metronidazole, Ampicillin
Bacteroides bivius	Clindamycin, Metronidazole, Ampicillin
Citrobacter freundii	Third-generation Cephalosporin
Rhodotorula rubra	Amphotericin B, with or without Fluconazole

Patients with alligator bites should also undergo radiographic evaluation to check for underlying complications like bone fractures or retained foreign bodies [8,10]. Assessing and restoring potential damage to vessels, nerves, or tendons is crucial, as injuries of this nature may lead to limb ischemia secondary to vascular damage or compression [15].

Beyond the physical repercussions, alligator bites can lead to enduring consequences, including disfigurement and psychological trauma. Namely, post-traumatic stress disorder can occur due to the atypical and surprising nature of the attack [14]. The psychological impact of large animal attacks has been insufficiently documented, warranting close follow-up and support for affected patients, as injuries of this matter may be more than skin-deep [14]. Future research investigating the long-term outcomes and psychological effects on alligator bite survivors could offer valuable insights into the broader repercussions of these incidents.

It is also important to highlight patient's autonomy and how medical providers must respect the decisions of each patient regardless if it is believed to be in their best interest to treat. This patient deferred treatment by not receiving sutures in a timely manner as well as refusing antibiotics. Thankfully, his injuries were not major and he did not develop an infection. This is an excellent example of how clinicians should always involve patients in clinical decision making.

Given the predatory nature of alligators and other crocodilian species, coexistence necessitates vigilance and adherence to safety guidelines. To avert adverse encounters, it is advisable for humans to maintain a considerable distance from alligators when encountering them in natural settings, even if the intention is observation or photography [16]. Additionally, near bodies of fresh or brackish water, individuals should abstain from feeding alligators or discarding food remnants in the water [16]. These measures are pivotal in sustaining a secure environment for both humans and alligators to share. Future endeavors investigating the efficacy of preventive measures, such as public education campaigns and habitat management strategies, may help to develop more targeted approaches to reduce human-alligator interactions and foster safety in regions with substantial alligator populations.

## Conclusions

In conclusion, encounters with crocodilian species pose a concern for human safety in the southern United States, particularly in states like Florida with significant alligator populations. Despite their ecological importance and conservation efforts, alligator attacks on humans can result in severe injuries and even fatalities. Prompt and appropriate medical management is crucial for mitigating the potential complications associated with alligator bites, including hemorrhage, infection, and tissue necrosis. Preventive strategies, including maintaining a safe distance from alligators and refraining from feeding these reptiles, are essential for ensuring a harmonious coexistence.
